# Supplementary material for: Synthetic ferrimagnet nanowires with very low critical current density for coupled domain wall motion
Source: Sci Rep. 2017 May 9;7:1640. doi: 10.1038/s41598-017-01748-7 (PMC5431626; doi:10.1038/s41598-017-01748-7)
Supplement: Supplementary file 1 — Supplementary Information [file 41598_2017_1748_MOESM1_ESM.pdf]

Synthetic ferrimagnet nanowires with very low critical current density for coupled domain wall motion
Supplementary Information

Serban Lepadatu, Henri Saarikoski, Robert Beacham, Maria Jose Benitez Romero, Thomas A. Moore, Gavin Burnell, Satoshi Sugimoto, Daniel Yesudas, May C. Wheeler, Jorge Miguel, Sarnjeet S. Dhesi, Damien McGrouther, Stephen McVitie, Gen Tatara, & Christopher H. Marrows

March 27, 2017

In this document we present supplementary information on five topics: (i) we describe the FMR measurements used to determine the Gilbert damping constant α; (ii) we discuss in more detail the DW structures in SAFs and SyFs; (iii) we give additional information about the magnetoresistance in our SyF samples; (iv) we describe in more detail the one-dimensional model used to describe the depinning of the coupled DWs; and (v) we describe supplementary simulations that we have carried out using that model to study the effect of varying different physical parameters on the behaviour of the system.

S.1 Ferromagnetic resonance measurements

In order to make a measurement of the damping parameter in a realistic structure, two further samples of Ru/$Co_{90}Fe_{10}$/Ru were grown according to the same protocol as the multilayer SyF structures. The thicknesses of the CoFe layers were 10 nm and 13.3 nm. Magnetisation dynamics measurements were made using a vector network analyser ferromagnetic resonance (VNA-FMR) spectrometer operating at microwave frequency transmitted from a 50 Ω impedance matched waveguide (X-band 8-11 GHz) mounted within an electromagnet that can apply fields in the range −200 to +200 mT. A more detailed description of the experimental set-up, measurement protocol, and data analysis methods may be found elsewhere[1].

The variation of the ΔS_{21} parameter (defined as the transmission between the ports of the VNA subtracted from a polynomial background fit) for a series of field sweeps at different microwave excitation frequencies f are shown in Fig. S1(a) and (b) for the 10 nm and 13.3 nm CoFe films, respectively. Clear resonance peaks are observed, which move up in field and broaden as f rises. These were fitted with Lorentzian functions to determine the resonance field

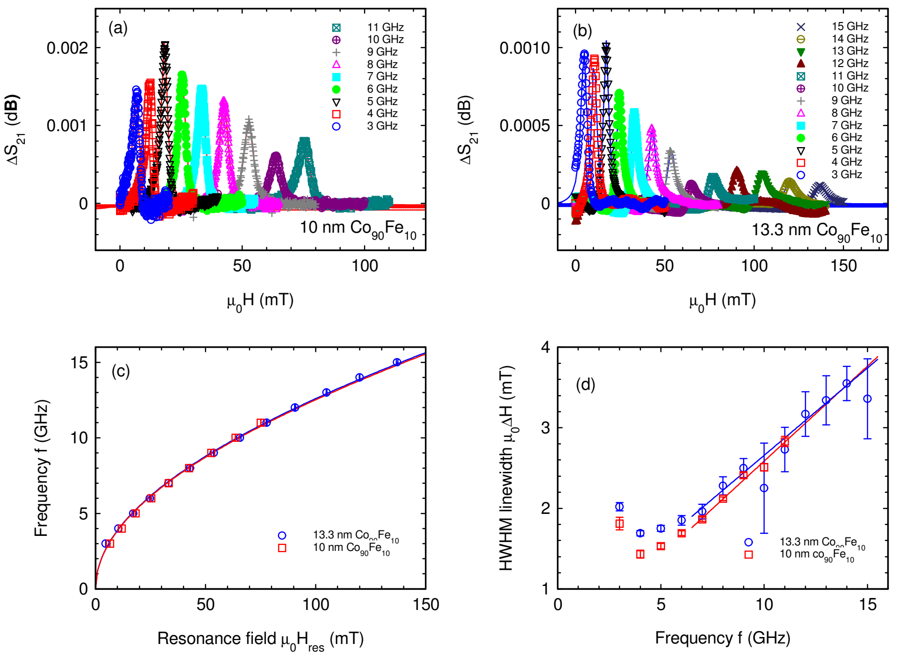

Figure S1 | **Ferromagnetic resonance measurements.** FMR peaks in the S_{21} parameter for **a,** the 10 nm CoFe film and **b,** the 13.3 nm CoFe film. Solid lines show the fitted Lorentzians. **c,** Kittel plots for the two films, with fits to the Kittel equation shown as solid lines. **d,** Frequency dependence of the linewidth $\Delta H(f)$ for the two films with straight lines of best fit for data where the magnetisation is saturated.

H_{res} and linewidth ΔH. These fits are shown as solid lines in Fig. S1(a) and (b).

Kittel plots showing the relationship between the f and H_{res} for the two films are shown in Fig. S1(c). The Kittel equation for a thin film with negligible magnetocrystalline anisotropy subject to an in-plane applied field was fitted to the data in both cases:

$$f = \left(\frac{\gamma}{2\pi}\right)\mu_0\sqrt{H_{\mathrm{res}}\left(H_{\mathrm{res}} + M_{\mathrm{eff}}\right)}, \qquad \text{(S1)}$$

where γ is the gyromagnetic ratio, μ_0 the permeability of free space, and M_{eff} is the effective magnetisation. The gyromagnetic ratio is related to the effective g-factor through the relation $\gamma = g_{\mathrm{eff}}\mu_{\mathrm{B}}/\hbar$, where μ_{B} is the Bohr magneton. Taking a stoichiometrically weighted average of the tabulated values of g_{eff} for Fe and Co[2], we obtained $g_{\mathrm{eff}} = 2.16$ for $Co_{90}Fe_{10}$. This gives the expected gyromagnetic ratio for our alloy composition as $\gamma/2\pi = 30.3$ GHz/T. (This is close to the value of $\gamma/2\pi = 31$ GHz/T reported by Rantschler *et al.* for a CoFe film of unspecified thickness and composition[3].) Fits of equation S1 to the data are shown as solid lines in Fig. S1(c). The values of effective magnetisation returned by the fits are given in table S1. The values are close the saturation magnetisation as measured by VSM, $M_{\mathrm{s}} = 1.4$ MA/m.

Table S1: FMR linewidth fitting parameters for the two CoFe films.

$Co_{90}Fe_{10}$ thickness (nm)	M_{eff} MA/m	$\mu_0\Delta H(0)$ (mT)	α
13.3	1.30 ± 0.01	0.5 ± 0.1	0.0066 ± 0.0004
10	1.28 ± 0.02	0.23 ± 0.02	0.0071 ± 0.0006

The FMR linewidth is a measure of the loss mechanisms associated with the precessional damping. The Gilbert damping constant α can be extracted from the frequency dependence of the linewidth according to the expression

$$\Delta H(f) = \Delta H(0) + \alpha \frac{f}{\gamma\mu_0}, \qquad \text{(S2)}$$

where $\Delta H(0)$ is due to the magnetic relaxation caused by the sample inhomogeneity. Plots of $\mu_0\Delta H(f)$ vs. f are shown in Fig. S1(d), where a clear linear dependence is observed for both the samples, but for minor deviations from linear behaviour at low frequencies arising from insufficient field to fully saturate the magnetisation. As a result, only data for $f > 5$ GHz were fitted. The results of these straight line fits of equation S2 are given in table S1. The two values just about agree to within one error bar of each other. On the basis of these results we took the value $\alpha = 0.007 \pm 0.001$ to model and analyse our data. This value is consistent with other measurements[3,4].

S.2 Wall structures

It is well-known that in patterned single-layer ferromagnetic wires the DW structure is strongly influenced by both the wire width and film thickness[5]. The competition between anisotropy, exchange and magnetostatic energies determines the type of DW, with symmetric transverse structures observed only for very narrow and thin wires, gradually changing into asymmetric transverse structures and finally into vortex structures as the wire width is increased. The magnetic stray field set-up by magnetic moments transverse to the wire, as is the case for a transverse DW, results in a significant increase in the magnetostatic energy, which is the origin of the so-called shape anisotropy[6]. As the wire width is increased, the uncompensated transverse demagnetising fields due to the magnetic dipole result in increasing distortions of the DW, lowering the magnetostatic energy at the cost of increased exchange energy. Thus for single-layer ferromagnetic wires the static DW structure is intrinsically coupled to the device dimensions[7].

When considering the DW dynamics, both field and current-driven, the picture becomes even more complicated, with DW transformations observed[8,9], and vortex-core mediated precessional motion for stronger driving forces[10] resulting in a complex dependence of threshold current densities on DW structure[11].

On the other hand for SAF structures, because the magnetic dipole set-up by transverse magnetic moments is compensated, the DW structure is effectively decoupled from the device dimensions. This results in narrow transverse DWs even in wide tracks, as we have observed using LTEM and XMCD-PEEM imaging, where vortices would be present in single ferromagnetic wires. For SyF

tracks, even though the transverse magnetic dipole is not fully compensated, the DW structure is largely determined by the antiferromagnetic coupling at the interfaces resulting in a decoupling from the track width. Narrow DWs of the Néel type are also observed in SyF tracks, as shown in Fig. 1d in the main text. It was shown previously that for antiferromagnetically coupled bilayers the DW width is largely determined by exchange and anisotropy energies, in the absence of an applied field, with values between 60 nm to 150 nm in Co/Ru/Co thin films[12]. For our SyF tracks the width of the injected DWs was measured using high-resolution TEM images to be ~ 100 nm with no dependence on track width. Micromagnetics simulations of DWs in SyF tracks confirm the symmetric transverse structure observed in the imaged samples. These narrow walls will present large magnetisation gradients to spin-polarised currents that pass through them.

S.3 Magnetoresistance loop

Typical magnetoresistance (MR) measurements are shown in Fig. 4a for a 400 nm wide SyF track, measured at a low current density. A complete hysteresis loop was also measured and the main magnetisation states are summarised in Fig. S2. There are two main contributions to the MR in these samples, namely giant magnetoresistance[13] (GMR) and anisotropic magnetoresistance[14] (AMR). GMR results in the lowest resistance state when the two $Co_{90}Fe_{10}$ layers are parallel and the highest resistance state when the layers are anti-parallel. On the other hand the AMR contribution is lowest when the current direction is perpendicular to the local magnetisation direction, as is the case for transverse magnetisation, and highest when the current is parallel to the magnetisation direction. The thick layer reverses only once, around zero field where the net moment switches. Meanwhile, the thin layer switches three times, at zero field when the net moment reverses, but also twice at stronger fields, where the system undergoes transitions between ferrimagnetic and saturated ferromagnetic states. We track this process, and its effects on the measured resistance, as follows.

The GMR contribution is larger than the AMR contribution, thus the resistance takes its lowest value in states A and E when the layers are fully saturated. The gradual transition from state A to state B (remanence), characterised by monotonically increasing resistance (see red curve in Fig. S2), is due to the gradual rotation of magnetisation in the thinner $Co_{90}Fe_{10}$ layer as the antiferromagnetic coupling competes with the applied field, increasing the GMR contribution to the resistance. Because the magnetisation in the ellipse rotates more quickly with decreasing field magnitude as compared to the track section, 360° DWs are trapped in the thinner $Co_{90}Fe_{10}$ layer, stabilised by the antiferromagnetic coupling to the thicker $Co_{90}Fe_{10}$ layer. Formation of 360° DWs was observed in LTEM imaging of magnetisation reversal and is also reproduced by micromagnetics simulations as shown in state B in Fig. S2.

Because of the combined negative AMR and GMR contribution of 360° DWs a further increase in the sample resistance state is achieved by removal of these DWs using either a minor field loop or (as we shall see below) by spin-transfer torque from current pulses, reaching the highest resistance state in the fully anti-parallel magnetisation state D_0, which is the zero-field ground state of the

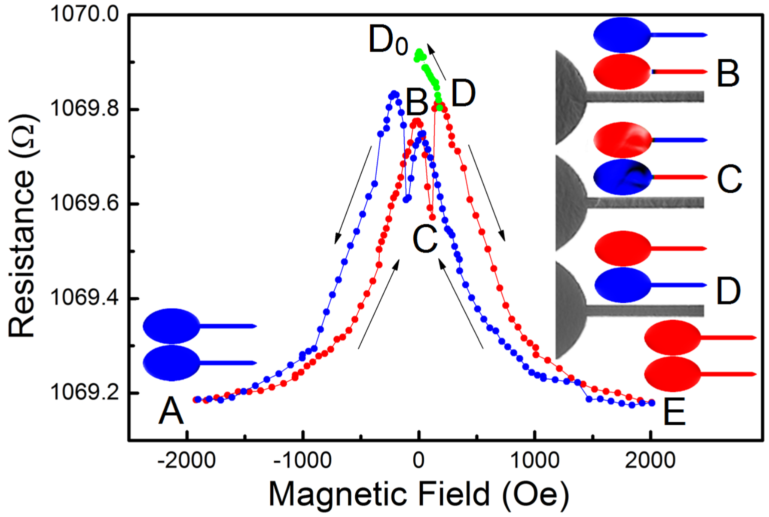

Figure S2 | Full magnetoresistance hysteresis loop of a SyF nanowire. Longitudinal MR measurement of a patterned SyF sample with a 400 nm wide wire and micromagnetics simulations showing some of the main magnetisation states. In this case $t_1 = 13.3$ nm and $t_2 = 6.6$ nm. The micromagnetics simulations show both the thick (top) and thin (bottom) $Co_{90}Fe_{10}$ layers for states B to E, accompanied by LTEM images on separately imaged structures (with an 800 nm wide wire) for states B-D. State D_0 is the fully anti-parallel ground state of the system, which can be reached by following the trajectory marked with green points. The labelled states are described in the text. Lorentz micrographs of a different 800 nm wide wire (but with the same layer thicknesses) prepared on a silicon nitride membrane are shown for comparison.

system.

The minor field loop leading from state B to state D_0 is explained first. From state B, increasing the applied magnetic field gradually rotates the thicker $Co_{90}Fe_{10}$ layer towards the field direction with the thinner $Co_{90}Fe_{10}$ layer rotating away from the field direction due to the antiferromagnetic coupling. This results in decreasing resistance due to the decrease in GMR contribution and introduction of negative AMR contribution. The magnetisation in the elliptical element rotates more quickly due to the smaller coercivity, resulting in formation of a 180° DW at the base of the track and removal of the 360° DW as shown in state C in Fig. S2, Fig. 1 and Fig. 2. Further increasing the field drives the 180° DW along the track, switching its magnetisation direction until state D is reached. As the magnetisation in the thicker $Co_{90}Fe_{10}$ layer lies along the field direction now, with the thinner $Co_{90}Fe_{10}$ layer coupled anti-parallel, the removal of both the negative AMR and GMR contributions results in a higher resistance state for the magnetisation state D. Relaxing back to zero field achieves the highest resistance state in D_0 where the contribution from 360° DWs is removed. D_0 is the ground state of the system, where full antiparallel alignment of the layer moments is achieved at zero applied field. Increasing the field from state D onwards gradually rotates the magnetisation in the thinner $Co_{90}Fe_{10}$

layer towards the field direction, characterized by decreasing resistance, as the applied field overcomes the antiferromagnetic coupling, eventually saturating the device in state E (equal but opposite to state A).

Another method of removing the 360° DWs consists of applying current pulses in state B. Here the sample is brought to remanence (state B) after saturation in state A and a single current pulse with current density -10^{11} Am^{-2} (electron flow away from the elliptical pad) and duration 10 µs is applied. Following this, the resistance is measured to ascertain the magnetisation state. In all cases the resistance state jumps to that for state D indicating the removal of the 360° DW. Micromagnetics simulations of current-driven 360° DWs in the SyF geometry show that this magnetisation structure is unstable and is quickly annihilated by introduction of vortex/antivortex cores from the track edges.

S.4 1-D model and equations of motion

The DW dynamics in the 1-D model is calculated by solving the equations of motion for the two wall parameters for each wall, the positions of the walls along the wire Z_i, $i = 1, 2$ and the angles of the spins with respect to the wire plane at the center of the wall ϕ_i, $i = 1, 2$ (see Fig. 3a in the main text). We set $t_1 = 13.3$ nm and $t_2 = 6.6$ nm to match our experimental structures.

The DW width parameter is determined by the exchange stiffness A and the easy axis shape anisotropy K from $\Lambda = \sqrt{A/K}$. We set $\Lambda = 25$ nm in both layers based on realistic estimates of these two quantities described in the methods section. The magnetic hard axis is perpendicular to the wire plane and it determines the critical velocity of intrinsic pinning $v_\mathrm{c} = K_\perp \Lambda S/(2\hbar)$ where $K_\perp$ is the hard axis anisotropy energy and S is spin[15]. The velocity of the driving spins is $v_\mathrm{e} = Pa^3 j/(2eS)$, where j is the electrical current density, $P = 0.34$ is the spin polarisation of the current, a is the lattice constant, and e is the electron charge.

Pinning of a DW against spin transfer torque can be either intrinsic or extrinsic[16]. Estimating the intrinsic pinning current density from the expression $J_\mathrm{intrinsic} = \mu H_\mathrm{w}/\xi$ we find that the values are of the order of 10^{14} Am^{-2}. Thus, the pinning that is being overcome is clearly extrinsic in nature, caused by defects (*e.g* lithographic edge roughness) that provide local potential wells for the coupled domain-wall pair. We model the extrinsic pinning potential as a parabolic potential well with pinning strength k_i and width ξ_i. The pinning potential is either in only one of the layers ($k_2 = 0$) at position $Z_1 = 0$, *i.e.*, at the center of the initial wall position, or in both layers having pinning strengths k_1 and k_2 and positions $Z_1 = 0$ and $Z_2 = \ell$, respectively. The former case is shown in the main text in Fig. 3b where we have set the pinning strength k_1 equivalent to a 150 Oe field and width of the well equal to the wall width $\xi_1 = \Lambda$. The latter case is discussed below.

The equations of motion for the wall parameters are derived in Ref. 17. In dimensionless units they can be written as

$$\dot{Z}_+ + \alpha\Lambda\dot{\phi}_- = -v_c \cos 2\phi_+ \sin 2\phi_- + v_\mathrm{e} \\ - u(Z_-)\big(\mu_+\Delta_+ \sin(2\phi_-) + \mu_-\Delta_- \sin(2\phi_+)\big), \quad \text{(S3)}$$

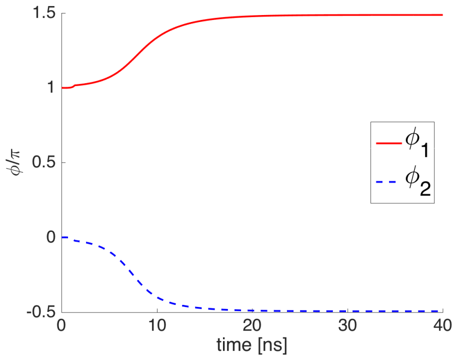

Figure S3 | The angle of the spins with respect to the wire plane at the center of the wall ϕ_i as a function of time. The calculation is performed in a regime where the interlayer coupling is very strong and the out-of-plane component is ferromagnetic $\Delta_\perp = -4\Delta_{||}$ where $\Delta_{||} = -2.77\ \mathrm{mJ/m^2}$. The current is turned on at $t = 0$. The spins are parallel and pointing out of plane after the time has evolved for about 20 ns.

$$\begin{aligned}\dot{\phi}_- - \alpha\frac{\dot{Z}_+}{\Lambda} + \frac{\beta}{\Lambda}v_\mathrm{e} = \\ &\frac{k_1}{2}(Z_+ + Z_- - \ell)\theta(\xi_1 - |Z_+ + Z_- - \ell|) + \\ &\frac{k_2}{2}(Z_+ + Z_-)\theta(\xi_2 - |Z_+ + Z_-|) - \\ &\frac{\mu_-}{2} \times \left[\Delta_{\|}w'(Z_-) + u'(Z_-)\left(\Delta_+\cos 2\phi_- + \Delta_-\cos 2\phi_+\right)\right], \end{aligned} \quad \text{(S4)}$$

$$\begin{aligned}\dot{Z}_- + \alpha\Lambda\dot{\phi}_+ = &-v_c \sin 2\phi_+ \cos 2\phi_- \\ &- u(Z_-)\big(\mu_-\Delta_+ \sin 2\phi_- + \mu_+\Delta_- \sin 2\phi_+\big), \end{aligned} \quad \text{(S5)}$$

$$\begin{aligned}\dot{\phi}_+ - \alpha\frac{\dot{Z}_-}{\Lambda} = &\frac{k_1}{2}(Z_+ + Z_- - \ell)\theta(\xi_1 - |Z_+ + Z_- - \ell|) - \\ &\frac{k_2}{2}(Z_+ + Z_-)\theta(\xi_2 - |Z_+ + Z_-|) - \\ &\frac{\mu_+}{2} \times \left[\Delta_{\|}w'(Z_-) + u'(Z_-)\left(\Delta_+\cos 2\phi_- + \Delta_-\cos 2\phi_+\right)\right], \end{aligned} \quad \text{(S6)}$$

where $Z_\pm = \frac{1}{2}(Z_1 \pm Z_2)$, $\phi_\pm = \frac{1}{2}(\phi_1 \pm \phi_2)$. The interlayer coupling constants (including both indirect exchange and magnetostatic contributions) are given by $\Delta_\pm \equiv \frac{1}{2}(\Delta_{\|} \pm \Delta_\perp)$, and $\mu_\pm = \frac{1}{2}(\mu_1 \pm \mu_2)$, where μ_i denotes the number of spins in the plane which is proportional to the layer thickness. The parameters in the model include the Gilbert damping parameter α and nonadiabatic torque coefficient β. We assumed long range interlayer coupling as described by the

functions

$$u(Z) \equiv \int_{-\infty}^{\infty} \frac{1}{\cosh z\ \cosh(z-2Z)}\ dz = 2Z\,\mathrm{csch}\,Z, \tag{S7}$$

$$w(Z) \equiv \int_{-\infty}^{\infty} \left(1 - \tanh z\ \tanh(z-2Z)\right)\ dz = 2Z \coth Z. \tag{S8}$$

For the solution of equations of motion (S3)–(S6) we used a numerical integration using a Runge-Kutta-Fehlberg 4th order method with a 5th order error estimator for the adaptive step size. The initial conditions for the walls at time $t = 0$ correspond to the ground state $Z_i(t = 0) = 0$, $i = 1, 2$ and $\phi_1(t = 0) = \pi$, $\phi_2(t = 0) = 0$.

Nonadiabatic driving by a spin-polarised current forces the spins at the center of the wall out-of-plane. The energies of the DWs are then lowered due to the ferromagnetic out-of-plane component of the interlayer coupling $\Delta_\perp > 0$. In the limiting case of a very strong interlayer coupling the orientation of spins at the center of walls are parallel and pointing nearly out-of-plane (see Fig. S3).

In the regime of viscous flow (low driving current density above the threshold current), the DWs move at velocity $v = (\beta/\alpha)v_{\mathrm{e}}$ (see Ref. 15). Walker breakdown[18] occurs at high current densities and results in deviations from this linear relationship between the spin velocity and wall velocity. The current density at the Walker breakdown depends strongly on the interlayer coupling and gives rise to the reduced DW velocity displayed in the center of Fig. 3b. The nature of the DW dynamics depends on the range of the interlayer coupling. We have used a long-range coupling which has a range of the order of DW width according to Eqs. (S7) and (S8), but qualitatively similar results are expected for a shorter interaction range.

S.5 Supplementary simulations

We discuss in this section supplementary simulations of domain wall dynamics and calculations of threshold current densities in different nanowire configurations within the 1-D model. The results show threshold current reduction in response to changes in the anisotropic coupling as the layer design is engineered, as discussed in the main text.

S.5.1 Form of pinning potential

The main text discussed simulations of domain wall velocity with a pinning potential situated in one of the layers. Simulations with a equal pinning potential in both planes reveal a higher threshold current consistent with stronger overall pinning. However, the threshold current decreases with interlayer coupling analogously. Fig. S4 shows the threshold current in the case of a pinning potential in both layers ($k_1 = k_2$, $\xi_1 = \xi_2 = \Lambda$, and $\ell = 0$). The pinning strength corresponds here to 20 times the pinning strength in Fig. 3a in the main text (3000 Oe). We note almost linear dependence on the interlayer coupling $\Delta_{||}$ in the regime of high $\Delta_{||}$, and that the threshold current density can still be driven to zero for sufficiently strong out-of-plane coupling even for this very string extrinsic pinning.

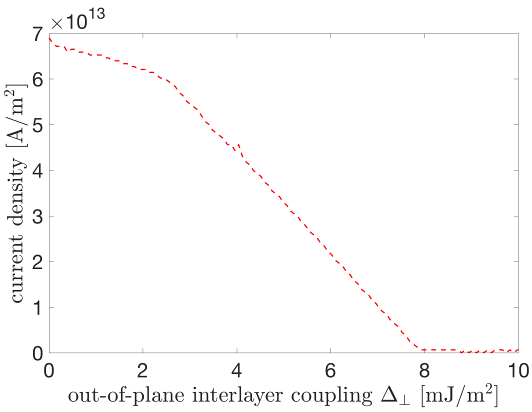

Figure S4 | Threshold current in the case of a strong pinning potential which is located at the same position in both layers. The calculation is performed in a regime where the out-of-plane component of the coupling is ferromagnetic $\Delta_\perp = -4\Delta_{||}$.

S.5.2 Magnitude of coupling anisotropy

Whilst in the main text we have performed our modelling assuming that the ratio of $\Delta_\perp/\Delta_{||} = -4$, which is close to that for our experimental system, it is instructive to consider how varying this ratio affects the behaviour of the system. We have therefore carried out further simulations of the threshold current density where the ratio $\Delta_\perp/\Delta_{||}$ is adjusted. The results are shown in Fig. S5 as a function of in-plane coupling strength.

At $\Delta_\perp = -\Delta_{||}$ there is no energy benefit when the spin transfer torques turn the values of ϕ_1 and ϕ_2 to out-of-plane and so the physical mechanism of threshold current reduction does not work in this case. As a consequence, the threshold current remains unaffected by the interlayer coupling. The same result applies also if the out-of-plane interlayer coupling strength is *weaker* than the in-plane coupling strength $|\Delta_\perp| < |\Delta_{||}|$. At higher out-of-plane interlayer coupling strength the threshold current decreases monotonically with out-of-plane coupling strength via the mechanism described in the main text.

It is therefore beneficial to engineer the coupling anisotropy to be as large as possible for the lowest critical current density. In our experiments the antiferromagnetic exchange coupling $\Delta_{||}$ is estimated at -0.75 mJ/m^2 when averaged over the wall structure. Using this figure calculations shown in Fig. S5 indicate that within the one-dimensional model the threshold current drops to zero if coupling anisotropy is around $\Delta_\perp/\Delta_{||} \approx -8$. This anisotropy is higher than the actual estimate of -4 for the experimental configuration and consistent with the fact that the observed threshold current is higher than zero.

Two-dimensional effects such as deformation of the domain wall, as demonstrated in LTEM images in Fig. 2 of the main text, and the spatial structure of the pinning potentials changes quantitatively our simpler one-dimensional picture. Moreover, the in-plane and out-of-plane couplings depend on the chosen geometry in the layer structure. In our case the strongest out-of-plane coupling would occur for a balanced SAF with equal thickness of the two layers, as we

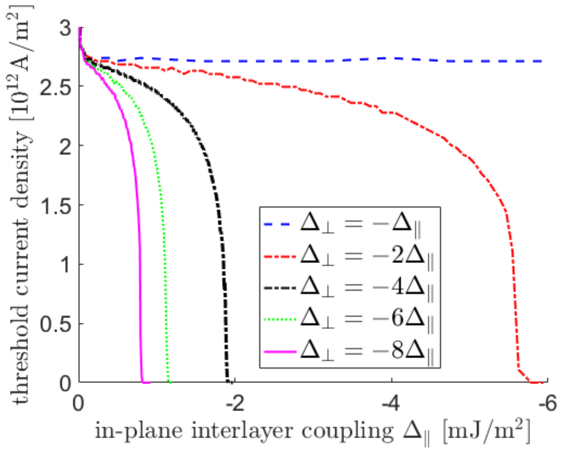

Figure S5 | Threshold current for various anisotropies of the interlayer coupling strength. The figure shows the threshold current density as a function of in-plane interlayer coupling at diffrent out-of-plane coupling constants. The threshold current decreases with increasing anisotropy.

shall discuss in more detail below. However, these details are not important for the qualitative understanding of the unpinning process and we conclude that the one-dimensional model captures the essential physics of the large threshold current reduction. We leave threshold current calculations with two or three-dimensional modelling of the system for future refined studies.

S.5.3 Layer structure design

As discussed in the main text and in the preceding section, the threshold current density depends crucially on the anisotropy of interlayer coupling strength. This in turn depends on the design of the nanowire. We studied theoretically different layer designs: a fully spin-compensated SAF design with equal layer thickness and SyF configurations with partial spin compensation. Micromagnetic simulation were performed to calculate the in-plane and out-of-plane interlayer coupling constants that consist on the exchange coupling and the magnetostatic couplings in a wire with constant total thickness of magnetic materials $t_1 + t_2 = 20$ nm, but with this total distributed into the two layers in different proportions. (In our experimental system, the ratio $t_2/t_1 = 1/2$. Since our model is symmetric with respect to layer label, this is completely equivalent to $t_1/t_2 = 1/2$.) The results of these simulations are summarised in Table S2 and show that the anisotropy is largest for a fully compensated SAF structure.

We performed additional simulations of domain wall dynamics with the one-dimensional model using the layer configuration and interlayer couplings shown in Table S2. We see from the calculated coupling parameters that by changing the thickness ratio of the two layers t_1/t_2 the out-of-plane interlayer $\Delta_\perp$ coupling is affected most, whilst the effect on the in-plane coupling $\Delta_{||}$ is not so strong. The largest out-of-plane coupling strength 3.55 mJ/m^2 occurs for a compensated SAF with equal thickness for the layers $t_1 = t_2$.

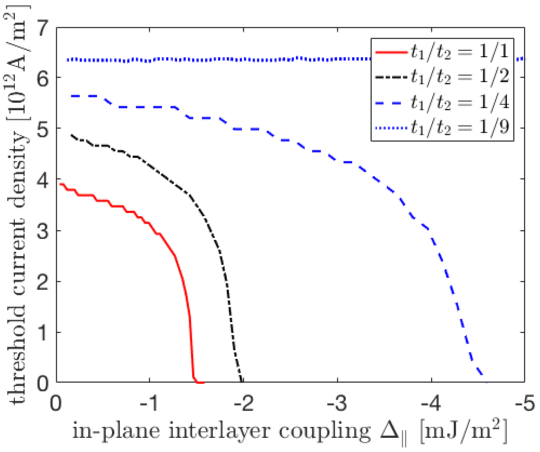

Figure S6 | Simulated threshold current in different nanowire layer structures. The figure shows the threshold current density as a function of in-plane interlayer coupling at layer thickness ratios t_1/t_2; a compensated SAF with equal layer thickness $t_1 = t_2$ and non-balanced SyFs with $t_1 < t_2$.

The threshold current densities calculated at thickness ratios of 1, 1/2, 1/4 and 1/9 are shown in Fig. S6. The pinning potential in the calculations is located in the thicker layer. The highest imbalance 1/9 leads to very low out-of-plane coupling in comparison to in-plane coupling $|\Delta_\perp| < |\Delta_{||}|$. Therefore the threshold current is not reduced by the interlayer coupling in the 1D model as discussed above, and this system effectively behaves as a single layer magnetic nanowire.

The threshold current at weak interlayer coupling decreases as the system becomes more balanced, since the out-of-plane coupling becomes stronger and the layer with pinning potential gets thinner. However, the interlayer coupling where the threshold current drops to zero is determined by the out-of-plane coupling strength. These calculations indicate that the threshold current depends strongly on the nanowire design. Therefore careful optimization of the layer structure is needed for practical device applications.

Table S2: Interlayer coupling strength from micromagnetic simulations. The in-plane $\Delta_{||}$ and out-of-plane $\Delta_\perp$ coupling strengths and their ratio are calculated for different layer thicknesses for a nanowire with total thickness $t_1 + t_2 = 20$ nm. The highest degree of coupling anisotropy occurs for a compensated SAF structure where $t_1 = t_2$.

Layer structure (t_1/t_2 ratio)	$\Delta_{\|\|}$ mJ/m^2	$\Delta_\perp$ mJ/m^2	$\Delta_\perp/\Delta_{\|\|}$
1/1	-0.768	3.55	-4.62
1/2	-0.750	3.02	-4.02
1/4	-0.715	1.91	-2.67
1/9	-0.670	0.609	-0.909

References

[1] Wheeler, M. C. *Manipulation of fullerene C_{60} spintronic devices via ferromagnetic resonance.* Ph.D. thesis, University of Leeds (2014).

[2] Coey, J. M. D. *Magnetism and Magnetic Materials* (Cambridge University Press, Cambridge, 2010).

[3] Rantschler, J., Ding, Y., Byeon, S.-C. & Alexander Jr., C. Microstructure and damping in FeTiN and CoFe films. *J. Appl. Phys.* **93**, 6671 (2003).

[4] Schoen, M. A. W. *et al.* Ultra-low magnetic damping of a metallic ferromagnet. *Nature Physics* **12**, 839 (2016).

[5] McMichael, R. D. & Donahue, M. J. Head to head domain wall structures in thin magnetic strips. *IEEE Trans. Magn.* **33**, 4167 (1997).

[6] Schrefl, T., Fidler, J., Kirk, K. & Chapman, J. Domain structures and switching mechanisms in patterned magnetic elements. *J. Magn. Magn. Mater.* **175**, 193 (1997).

[7] Nakatani, Y., Thiaville, A. & Miltat, J. Head-to-head domain walls in soft nano-strips: a refined phase diagram. *J. Magn. Magn. Mater.* **290-291**, 750 (2005).

[8] Kläui, M. *et al.* Direct observation of domain-wall configurations transformed by spin currents. *Phys. Rev. Lett.* **95**, 026601 (2005).

[9] Vanhaverbeke, A., Bischof, A. & Allenspach, R. Control of domain wall polarity by current pulses. *Phys. Rev. Lett.* **101**, 107202 (2008).

[10] Heyne, L. *et al.* Geometry-dependent scaling of critical current densities for current-induced domain wall motion and transformations. *Phys. Rev. B* **80**, 184405 (2009).

[11] Lepadatu, S. *et al.* The increase of the spin-transfer torque threshold current density in coupled vortex domain walls. *J. Phys.: Condens. Matter* **24**, 024210 (2012).

[12] Li, Z. *et al.* Magnetization precession and domain-wall structure in cobalt-ruthenium-cobalt trilayers. *J. Appl. Phys.* **109**, 07C113 (2011).

[13] Baibich, M. N. *et al.* Giant magnetoresistance of (001)Fe/(001)Cr magnetic superlattices. *Phys. Rev. Lett.* **61**, 2472 (1988).

[14] McGuire, T. R. & Potter, R. I. Anisotropic magnetoresistance in ferromagnetic 3*d* alloys. *IEEE Trans. Magn.* **11**, 1081 (1975).

[15] Tatara, G., Kohno, H. & Shibata, J. Microscopic approach to current-driven domain wall dynamics. *Phys. Rep.* **468**, 213 (2008).

[16] Tatara, G. & Kohno, H. Theory of current-driven domain wall motion: Spin transfer versus momentum transfer. *Phys. Rev. Lett.* **92**, 086601 (2004).

[17] Saarikoski, H., Kohno, H., Marrows, C. H. & Tatara, G. Current-driven dynamics of coupled domain walls in a synthetic antiferromagnet. *Phys. Rev. B* **90**, 094411 (2014).

[18] Schryer, N. L. & Walker, L. R. The motion of 180° walls in uniform dc magnetic fields. *J. Appl. Phys.* **45**, 5406 (1974).
